# Exploring the Smoking-Epilepsy Nexus: a systematic review and meta-analysis of observational studies: Smoking and epilepsy

**DOI:** 10.1186/s12916-024-03307-0

**Published:** 2024-03-04

**Authors:** Yerin Kang, Sieun Kim, Yunah Jung, Dai Sik Ko, Hyun-Woo Kim, Jung-Pil Yoon, Sunghwan Cho, Tae-Jin Song, Kihun Kim, Eunjeong Son, Yun Hak Kim

**Affiliations:** 1 School of Medicine, Pusan National University, Yangsan, Republic of Korea; 2 Division of Vascular Surgery, Department of General Surgery, Gachon University Gil Medical Center, Incheon, Republic of Korea; 3 Department of Neurology, Pusan National University Yangsan Hospital, Yangsan, Republic of Korea; 4 Department of Anesthesia and Pain Medicine, Pusan National University Yangsan Hospital, Yangsan, Korea; 5 Department of Surgery, Pusan National University Yangsan Hospital, Yangsan, Republic of Korea; 6 Department of Neurology, Seoul Hospital, Ewha Womans University College of Medicine, Seoul, Republic of Korea; 7 Department of Biomedical Informatics, School of Medicine, Pusan National University, Yangsan, Republic of Korea; 8 Department of Anatomy, School of Medicine, Pusan National University, Yangsan, Republic of Korea; 9 Division of Respiratory and Allergy, Department of Internal Medicine, Pusan National University Yangsan Hospital, Yangsan, Republic of Korea

**Keywords:** Smoking, Nicotine, Epilepsy, Seizure, Systematic review, Meta-analysis

## Abstract

**Background:**

Epilepsy, characterized by recurrent unprovoked seizures, poses significant challenges to affected individuals globally. While several established risk factors for epilepsy exist, the association with cigarette smoking remains debated. This study aims to conduct systematic review and meta-analysis to elucidate the potential association between smoking and the likelihood of epilepsy.

**Methods:**

The search was performed on March 31st, 2023, using the Medline, Embase, Web of Science, Scopus, and ScienceDirect. We included cohort, cross-sectional, and case–control studies in our meta-analysis, conducting subgroup analyses based on smoking history, sex, and epilepsy type to yield specific insights.

**Results:**

We identified 2550 studies, of which 17 studies were finally included in this study. The pooled odds ratio of epilepsy was 1.14 (0.96–1.36) in smokers compared to non-smokers. In current smokers compared to non-smokers, the odds ratio was 1.46 (1.13–1.89), while, in former smokers compared to non-smokers, the odds ratio was 1.14 (0.83–1.56).

**Conclusions:**

While the overall association between smoking and epilepsy did not reach statistical significance, a notable association was found among current smokers. The study emphasizes the importance of smoking cessation as a potential preventive measure against epilepsy, especially given the proconvulsive effects of nicotine. Future research should address limitations and explore specific clinical scenarios to enhance our understanding of the complex relationship between cigarette use and epilepsy.

**Systematic review registration:**

CRD42022342510.

**Supplementary Information:**

The online version contains supplementary material available at 10.1186/s12916-024-03307-0.

## Background

Epilepsy, defined as a neurological condition marked by two or more unprovoked seizures, is recognized by the international league against epilepsy [1]. These seizures, often unpredictable and sudden, disrupt daily life and interpersonal relationships, contributing to heightened cognitive challenges. Such challenges encompass issues like memory impairment, impaired executive functioning, and deficits in both verbal and nonverbal skills [2]. Globally, epilepsy impacts 70 million individuals, with an annual mortality rate of approximately 125,000 among those affected [3]. This condition imposes a significant economic burden, accounting for up to 1% of total national healthcare expenditure in many countries [4]. Several established risk factors contribute to the onset of epilepsy in adults, including head trauma, central nervous system (CNS) infections, various types of strokes, CNS malignancies such as cortically based tumors, Alzheimer’s disease, and other neurodegenerative conditions [5]. Specifically, identified modifiable risk factors are pregnancy, alcohol-related issues, depression or other psychiatric disorders, and injuries [6, 7].

Cigarette smoking, a prevalent global habit known for its high addictiveness and profound health implications, has been implicated as a potentially modifiable risk factor based on recent Mendelian randomization analysis [8]. However, despite numerous observational studies exploring the connection between smoking and unprovoked epilepsy, the association remains a subject of dispute. Some studies suggest an increased incidence of epilepsy in smokers [9, 10], whereas other investigations find no significant difference in epilepsy rates between smoking and non-smoking groups [11]. Moreover, findings regarding whether the risk of epilepsy decreases after quitting smoking vary [12, 13]. Despite the inconsistencies in the results of these related studies, there has been no systematic literature review or meta-analysis conducted to date. The primary aim of this study is to investigate the potential association between smoking and the likelihood of epilepsy.

## Methods

### Protocol and registration

Our study protocol was registered with PROSPERO. We followed the methodology accordance to the Preferred Reporting Items for Systematic Reviews and Meta-Analyses (PRISMA) guidelines.

### Eligibility criteria

We included studies examining the association between epilepsy and smoking. Cohort or case–control studies were eligible for inclusion in this study. Studies were excluded if the primary outcome involved provoked seizures or if the study population encompassed individuals previously diagnosed with epilepsy. Papers with duplicate databases or inclusion errors were also excluded. Reviews, abstracts, and editorial materials were excluded. The inclusion criterion for studies from the same center prioritized reports with a higher number of samples relevant to this study.

### Search strategy

On March 31st, 2023, an extensive systematic literature exploration was conducted across multiple medical databases, including Medline, Embase, Web of Science, Scopus, and ScienceDirect, to identify pertinent published articles. To formulate search strategies tailored to each database, the primary emphasis was placed on leveraging the MeSH term and associated entry terms for “smoking,” “epilepsy,” “case–control study,” and “cohort study.” The detailed search methodologies are outlined in Additional file 1: Supplementary Table 1, and these strategies were established through consensus among all co-authors. All searches were confined to human studies and articles.

### Study selection

Three authors (YK, SK., JY) independently conducted the literature search, evaluating the titles and abstracts of each study. The same authors also thoroughly reviewed the full-text articles that met the inclusion criteria. Any disagreements were resolved through discussion among the authors.

### Data extraction and statistical analysis

Data were extracted from the publications independently by three authors (YK, SK, JY), and the following information was recorded: title, abstract, first author, year of publication, country of publication. Through a full-text assessment, number of samples, study design, effect measures, and exposure category were additionally extracted.

### Statistical analyses

Odds ratios (ORs) and their 95% confidence intervals (CIs) were derived from the included studies through 2 × 2 contingency tables [14]. To assess the heterogeneity of the effect estimates, the *I*^2^ statistics classification by Higgins et al. (2003) was employed [15]. Heterogeneity was categorized as moderate (*I*^2^-value 50–75%) or considerable (*I*^2^-value > 75%), indicating significant heterogeneity [16]. If the heterogeneity exceeded 50%, the random effects method was used; otherwise, the fixed effects method was employed. Forest plots were generated to clearly illustrate the pooled ORs. Subgroup analysis was performed based on various factors, including smoking status, sex, study design, and type of epilepsy. To assess the impact of individual studies on overall effect measures, sensitivity analysis was conducted. Review Manager 5.4 software (Cochrane, U.K.) conducted all analyses.

### Risk of bias in individual studies

The risk of bias in the included studies was qualitatively assessed using the Newcastle–Ottawa scale, with the adapted version applied for cross-sectional studies. Study scores were then categorized into three levels of evidence: “good,” “fair,” and “poor,” following the standard set by the Agency for Healthcare Research and Quality.

### Publication bias

Publication bias was assessed visually through the creation of a funnel plot. Additionally, Egger’s regression test was employed to evaluate the statistical significance of any potential publication bias. All analyses for publication bias were performed using STATA 13 software (Stata Corporation, U.S.A.).

### Certainty assessment

We employed the Grading of Recommendations, Assessment, Development, and Evaluations (GRADE) approach to evaluate the quality of the evidence. The quality was graded as high, moderate, low, or very low, determined by the degree of confidence in the accuracy of the effect estimate based on eight factors.

## Results

### Study selection and characteristics

Initially, we identified a total of 2550 studies through comprehensive database searches. Following the exclusion of non-human, non-article, and review articles, 1662 records remained for further scrutiny, involving the assessment of titles and abstracts. Subsequently, 1505 papers were eliminated due to reasons such as duplication, review article classification (narrative or systematic), unavailability of full-text, and absence of quantitative data.

**Fig. 1:**
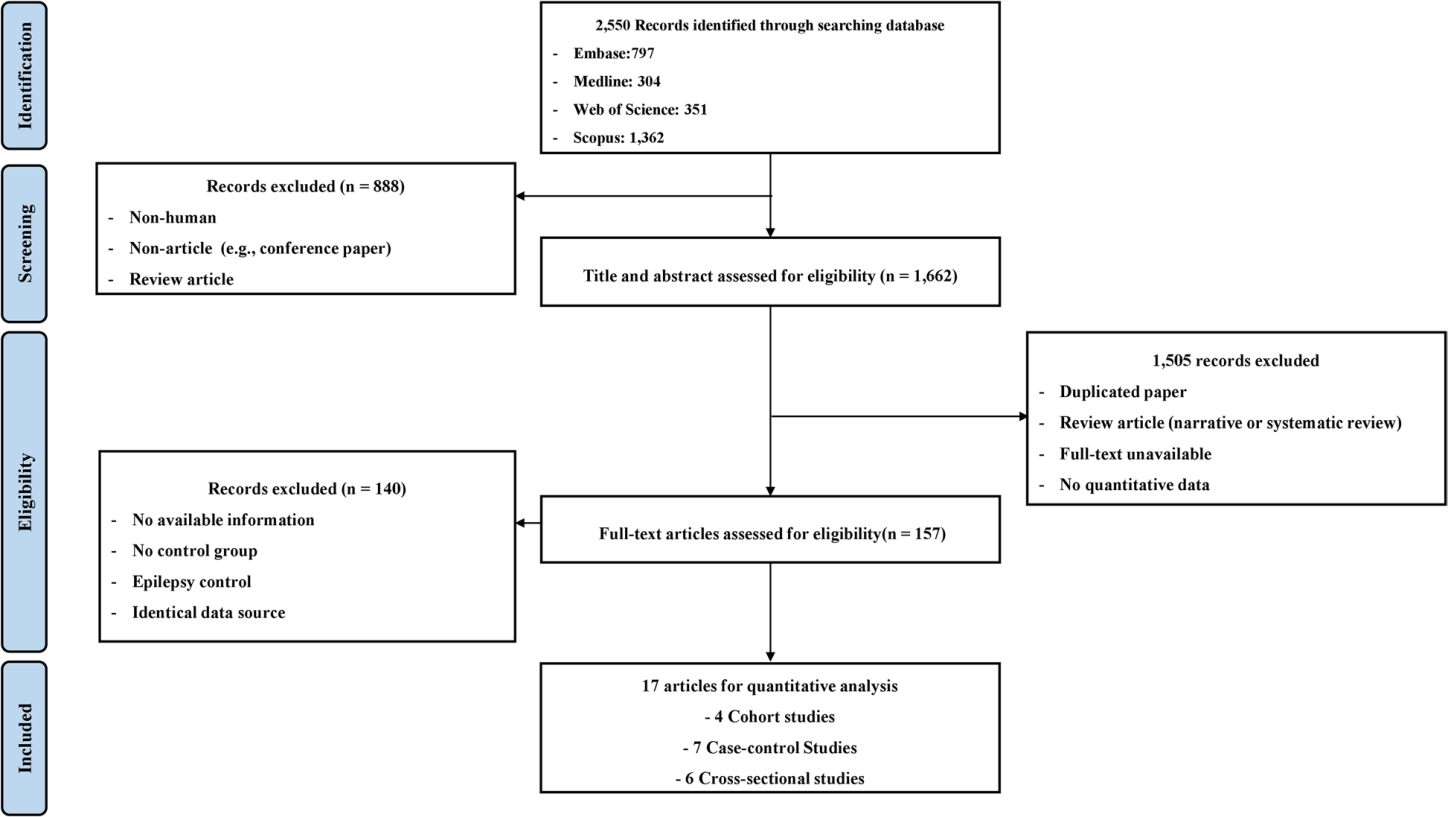
PRSIMA flow diagram for systematic reviews which included searches of databases

After this rigorous screening process, 157 studies underwent full-text review. Within this phase, papers lacking pertinent information, a control group, those related to epilepsy control, and those sharing identical data sources were excluded. Ultimately, 17 studies were deemed suitable for inclusion in the meta-analysis, as delineated in Fig. 1 [9–13, 17–28]. This selection comprised 4 cohort studies, 7 case–control studies, and 6 cross-sectional studies. Comprehensive details regarding the characteristics of these included studies can be found in Table 1.

**Table 1:** Characteristics of included studies

Authors	Place and time of study	Gender	Study designs	Number of samples	Exposure categories	Outcome
**Cohort**						
Gao (2008) [12]	U.K 2000–2005	Both	Cohort	419,747 Case number 131	Nonsmoker, ex-smoker, smoker; based on Read codes and AHD codes	Seizure and epilepsy
Hamidou (2013) [17]	France 1985–2010	Both	Cohort	4358 Case number103	Non-smokers, smokers (> 1 cigarette/day)	Seizure
Reiter (2013) [18]	Norway 2013	Female	Cohort	106,935 pregnancies Case 711	Smoking during pregnancy (women with epilepsy who did and did not use antiepileptic drugs)	Epilepsy
Johnson (2018) [19]	U.S., 1987–2013	Both	Cohort	15,792 Case 348 Controls 10,072	Smoking Never smoker, < 25 pack year, > = 25 pack year	With and Without Late Onset Epilepsy (based on ICD-9)
**Case–control**						
Cockerell (1996) [20]	U.K.,	Both	Case–control	Cases 123 Controls 133	Smoking amounts not specified	Inactive and active epilepsy
Janszky (2009) [21]	Sweden Male 1992–1993; Female 1992–1994	Both	Case–control	Cases 44 Controls 4023	Non-smokers. Ex-smokers (stopped smoking for more than 2 years), Smokers (currently smoking or stopped smoking within the previous 2 years)	Epilepsy
Borthen (2011) [22]	Norway 1999–2006	Female	Case–control	Cases 205 Controls 205	Smoking during pregnancy (yes/no)	Inactive and active epilepsy
Naldi (2013) [23]	Italy 2013	Both	Case–control	62 Case 33 Control 31	Non, current, former smoker (For former smokers only (*n* = 96/434): year in which the study was conducted minus the year of quitting smoking.)	Autosomal dominant nocturnal frontal lobe epilepsy patients
Im (2016) [10]	Korea 2016	Both	Case–control	3016 Case 180 Control 2836	Smoker, non-smoker	Epilepsy
Aguirre (2017) [24]	Spain 2013–2014	Both	Case Control	278 Case 85 Controls 193	Smoker, non-smoker, former smoker (based on survey)	Focal Epilepsy, Generalized Epilepsy
Wang (2021) [9]	Australia, 2004–2019	Both	Case–control	427 Case 40 Controls 387	Never, Current Smoker (defined as smoking within 12 months prior to recognition of cognitive decline)	With and Without Epilepsy (DSM-5)
**Cross-sectional**						
Kobau (2008) [13]	U.S., 2005	Both	Cross-sectional	120,327 Cases 2203	Smoking amounts not specified	Epilepsy
Svalheim (2013) [25]	Norway and Austria 2013	Both	Cross-sectional	291 Case 211 Control 80	Only Current Smoker	Epilepsy
Cui (2015) [26]	U.S 2010	Both	Cross-sectional	27,139 Case 480 Control 26,659	Non, current, former smoker (In the past 12 months, has a medical doctor, dentist, or other health professional advised you to quit smoking or quit using other kinds of tobacco)	Epilepsy
Tumay (2015) [11]	Turkey 2015	Both	Cross-sectional	202 Case 106 Control 96	Smoker, non-smoker (based on survey)	Epilepsy (Epilepsy duration)
Wang (2016) [29]	U.S 2016	Both	Cross-sectional	43,020 Case 604 Control 42,416	Smoker, non-smoker	Epilepsy
Stefanidou (2022) [28]	U.S 1991–1995	Both	Cross-sectional	2986 Case 55 Control 2931	Current smoker, non-smoker(self-report)	Incident Epilepsy, Without incident epilepsy (routine chart review, self-report, ICD-9)

*DSM* Diagnostic and Statistical Manual of Mental Disorders, *ICD* International Classification of Diseases, *ILAE* International League Against Epilepsy

### Synthesis of results

#### Overall result

**Fig. 2:**
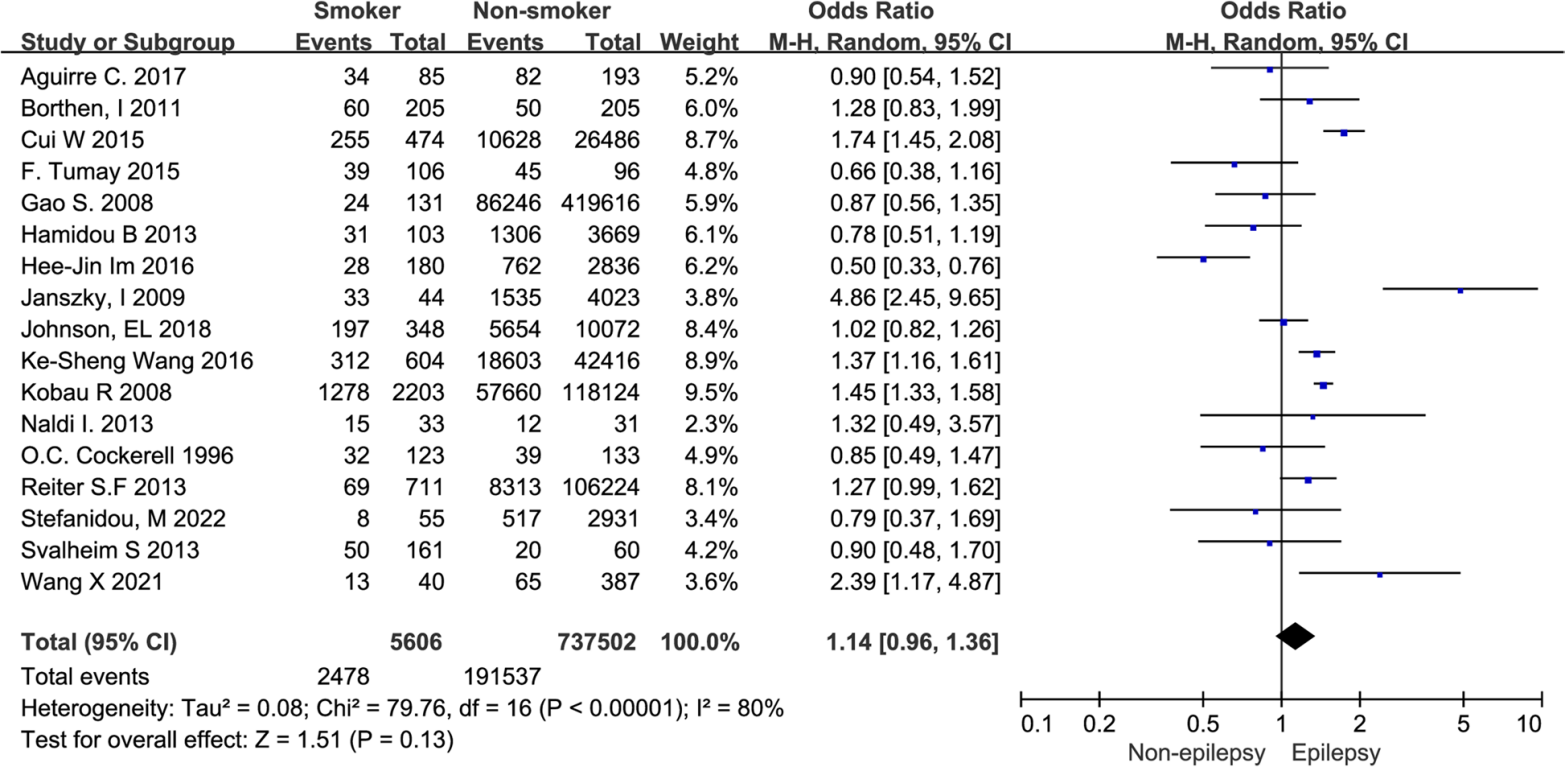
The forest plot depicting the pooled odds ratio of epilepsy in smokers compared to non-smokers

Our analysis encompassed seventeen studies involving a total of 743,108 subjects. The pooled OR for epilepsy among smokers, in comparison to non-smokers, was 1.14 (0.96–1.36), as illustrated in Fig. 2.

#### Subgroup analysis

A detailed examination across four categories—smoking status, sex, study design, and type of epilepsy—was conducted, and the outcomes are summarized in Table 2. For current smokers compared to non-smokers, the OR was 1.46 (1.13–1.89) (Additional file 2: Supplementary Fig. 1). In the case of former smokers compared to non-smokers, the odds ratio was 1.14 (0.83–1.56) (Additional file 2: Supplementary Fig. 2). Within the male group, the odds ratio was 0.75 (0.46–1.23) (Additional file 2: Supplementary Fig. 3), and in the female group, it was 1.15 (0.74–1.80) (Additional file 2: Supplementary Fig. 4). Regarding study design, the OR for cohort studies was 1.04 (0.90–1.20) (Additional file 2: Supplementary Fig. 5), for case–control studies, it was 1.29 (0.75–2.23) (Additional file 2: Supplementary Fig. 6), and for cross-sectional studies, it was 1.32 (1.10–1.58) (Additional file 2: Supplementary Fig. 7). Furthermore, the OR for active epilepsy was 1.59 (1.42–1.78) (Additional file 2: Supplementary Fig. 8), while for inactive epilepsy, it was 1.18 (0.77–1.80) (Additional file 2: Supplementary Fig. 9).

**Table 2:** Exploring the association between smoking and epilepsy through subgroup analyzes of included studies

Outcome	Number of studies (*n*)	Heterogeneity (%)	Odds ratio (95% confidence interval, *p*-value)
Smoking status			
Current smoker	6	80	1.46 (1.13–1.89, *p* = 0.004)
Former smoker	6	84	1.14 (0.83–.1.56, *p* = 0.43)
Sex			
Male	3	0	0.75 (0.46–1.23, *p* = 0.26)
Female	3	0	1.15 (0.73–1.81, *p* = 0.54)
Study design			
Cohort	4	38	1.04 (0.90–1.20, *p* = 0.63)
Case control	7	84	1.29 (0.75–2.23, *p* = 0.36)
Cross-sectional	6	69	1.32 (1.10–1.58, *p* = 0.002)
Epilepsy type			
Active epilepsy	4	0	1.59 (1.42–1.78,* p* < 0.001)
Inactive epilepsy	4	80	1.18 (0.77–1.80, *p* = 0.45)

#### Sensitivity analysis

Conducting a sensitivity analysis revealed that the exclusion of the study conducted by Im et al. (2016) resulted in a noteworthy alteration of the overall outcome (Additional file 2: Supplementary Fig. 10) [10]. The statistical significance of the remaining studies remained unaffected by this exclusion.

### Risk of bias within studies

Among the 4 cohort studies, three received a rating of “good,” and one was rated as “fair.” For the 7 case–control studies, three were assessed as “good,” while four were rated as “fair.” Among the 6 cross-sectional studies, four were designated as “good,” and two received a “satisfactory” rating. A comprehensive evaluation of the risk of bias is available in Additional file 1: Supplementary Table 2.

### Publication bias

**Fig. 3:**
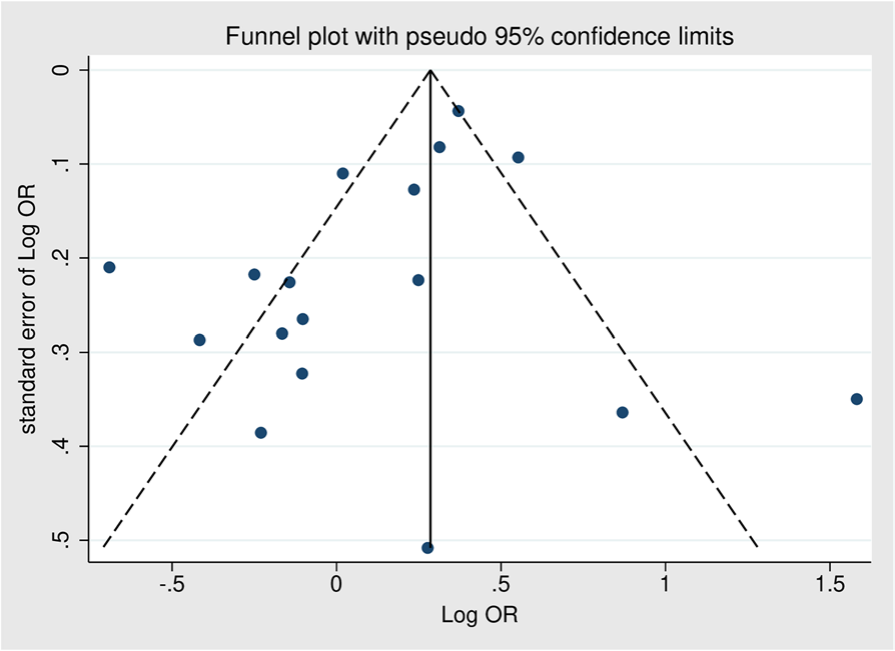
Funnel plot for evaluating the publication bias of overall outcome derived from 17 studies (*x*-axis: log odds ratio, *y*-axis: standard error of log odds ratio)

To visually evaluate publication bias regarding the overall OR of epilepsy, a funnel plot was constructed (Fig. 3). Subsequently, Egger’s regression test was conducted, indicating no significant evidence of publication bias (*p* = 0.102).

### Certainty assessment

The overall outcome underwent a comprehensive assessment across eight domains, and the quality of evidence was appraised using the GRADE approach. Following this evaluation, the quality of evidence for the overall outcome was categorized as very low, as depicted in Table 3.

**Table 3:** Certainty assessment of the overall analysis on smoking and epilepsy using the GRADE Approach

Outcomes	Certainty assessment							Effect	Certainty
	**No. of studies**	**Study design**	**Inconsistency**	**Indirectness**	**Imprecision**	**Publication bias**	**Other considerations**	**OR (95% CI)**	
Smoking—epilepsy	17	Serious^a^	Serious^b^	Not serious	Not serious^c^	Not serious^d^	No dose–response gradient Residual confounding, or biases Small effect size	1.14 (0.96–1.36)	Low

*OR* odds ratio, *CI* confidence interval

^a^All included studies are observational design

^b^Heterogeneity was 80%

^c^Very large samples size (over 4000) and *p* < 0.05

^d^According to Egger’s regression test (*p* = 0.102)

## Discussion

Numerous previous investigations have yielded conflicting findings on the relationship between smoking and epilepsy, with some studies suggesting an increased association [26, 30], while others report no discernible link [10, 29]. Given the divergent research outcomes, our study aims to clarify the definitive correlation between smoking and epilepsy. Our comprehensive meta-analysis revealed an OR of 1.14 (0.96–1.36) when comparing the occurrence of epilepsy in smokers to that in non-smokers. Notably, among current smokers, a significant correlation was evident, with an OR of 1.46 (95% CI 1.13–1.89). Although statistical significance eluded the overall association, a discernible trend implies a potentially elevated occurrence of epilepsy among smokers, particularly those who are currently smoking. This study addresses a crucial gap in the literature by synthesizing both historical and contemporary research on the association between smoking and epilepsy.

Although specific pathophysiological mechanisms through which chronic cigarette smoking influences the risk of seizures or epilepsy remain controversial [31], several plausible hypotheses have been proposed. One potential explanation for the heightened risk of epilepsy in smokers is its potential contribution to cerebral vessel atherosclerosis. This, in turn, may lead to neuronal impairment, accelerating the dysfunction of neuro-electrical networks and ultimately triggering epilepsy [9]. Another hypothesis suggests that, although a direct dose correlation between carbon monoxide-hemoglobin (CO-Hb) levels and the occurrence of seizures may not be evident, elevated CO-Hb levels observed in smokers could be associated with comorbidities, such as hypoxia, which may contribute to the manifestation of epilepsy [32]. In addition to nicotine, tobacco smoke, containing chemicals like arsenic, ammonia, and acetone has been shown in human and animal studies to possess the potential to induce seizures under specific conditions [32]. Additionally, tobacco smoke has been shown to modify the metabolism of various compounds processed by the cytochrome P450 and UDP-glucuronyl transferase systems [33, 34]. The compounds affected by this alteration may encompass medications or substances that either lower the seizure threshold or are antiseizure medications [35].

Significant insights emerged from a subgroup analysis examining the association between epilepsy and smoking status. The OR for individuals classified as current smokers revealed a heightened risk of epilepsy at 1.46 (1.13–1.89), emphasizing an increased risk associated with cigarette use. In contrast, former smokers exhibited an OR of 1.14 (0.83–1.56), suggesting a potential decrease in epilepsy risk after smoking cessation. These findings emphasize the importance of quitting smoking as a proactive measure to reduce the likelihood of developing epilepsy [13, 26]. They strengthen the validity of smoking cessation as a protective action against epilepsy, underscoring the potential benefits of quitting smoking for individuals concerned about this neurological condition. However, caution is warranted in addressing the various withdrawal symptoms associated with smoking cessation, particularly neurological symptoms like irritability, anger, frustration, anxiety, and depressed mood [36].

Upon scrutinizing the relationship between smoking and epilepsy stratified by sex, no significant findings were observed. The OR was 1.15 (0.74–1.80) for women and 0.87 (0.56–1.34) for men. Factors such as limited study participants, variations in the duration of exposure, and potential sex differences in the impact of smoking suggest that further investigation is needed to elucidate these distinctions.

In investigating the link between smoking and epilepsy concerning seizure activity, we identified an OR of 1.59 (1.42–1.78) for active epilepsy, signifying an elevated risk associated with smoking. Conversely, for inactive epilepsy, the OR was 1.18 (0.77–1.80), implying a less pronounced association. Individuals with active epilepsy, defined as those currently taking medication for the condition and experiencing seizures in the past year, underscore the importance of examining the efficacy of smoking cessation as a protective measure against epilepsy [37].

In assessing the impact of study design, we computed ORs for various research methodologies. Cohort studies yielded an OR of 1.04 (0.90–1.20), case–control studies produced an OR of 1.29 (0.75–2.23), whereas cross-sectional studies exhibited an OR of 1.32 (1.10–1.58), indicating a positive correlation. Cohort studies are commonly considered more robust due to their controlled parameters and extended follow-up periods, which serve to minimize bias and strengthen the association between exposure and disease. However, the scarcity of a sufficient number of cohort studies in our meta-analysis resulted in non-significant findings. Instead, the inclusion of more cross-sectional studies, primarily reliant on surveys, contributed to this outcome [18]. Due to these limitations, generalizing the analysis results became challenging. Therefore, to enhance the precision of future analyses, additional large-scale cohort studies conducted over extended periods within the general population are imperative.

## Limitations

Several studies included in our analysis were limited to patients with specific medical conditions. For instance, Janszky et al. (2009) exclusively focused on epilepsy in individuals with acute myocardial infarction [21]. This targeted approach may restrict the generalizability of our findings. Despite our intention to incorporate datasets encompassing unprovoked seizures, such as idiopathic and remote symptomatic seizures, while excluding induced seizures, we observed the inclusion of patients with various medical conditions, including withdrawal symptoms, sudden strokes, or other diseases. Consequently, the dataset is susceptible to selection bias, diminishing its representativeness for the general population. Furthermore, as this is a meta-analysis that synthesizes observational studies, it is challenging to infer causation.

The criteria for classifying epilepsy lacked uniformity across the included studies. Given the varied definitions of epilepsy among these studies, we relied on referencing the full-text methods and criteria to classify cases. This dependence on diverse criteria introduces variability and imprecision into the analysis. The process of obtaining adjusted ORs was hindered by the heterogeneity of adjusted variables across the studies. Each study employed different independent variables in their multivariate analyses through multiple regression. Consequently, the reliability of the overall adjusted OR may be compromised due to these variations in the adjustment process. Finally, limited data availability from the included studies precluded the conduct of subgroup analysis for the duration of exposure or dose–response analysis (pack-year).

## Future directions

While our study boasts strengths in executing diverse subgroup analyses, including those pertaining to tobacco history, sex, and epilepsy type, it is crucial to undertake further research to establish a definitive causal relationship between smoking and unprovoked seizures while addressing the study’s limitations. To achieve this, future investigations should prioritize data adjusted to account for these limitations. Instead of focusing solely on the frequency of seizures in patients with specific diseases, the emphasis should shift toward data collected from randomly selected epilepsy patients. Subsequent studies should delve into the risk of epilepsy in relation to cigarette use, enabling the confirmation of a dose–response relationship between cigarette consumption and epilepsy. The identification of a linear relationship between the control variable and the independent variable would provide greater clarity in establishing this connection.

## Conclusions

In conclusion, while our meta-analysis indicated that the overall correlation was not statistically significant, a discernible association was observed among current smokers. Further research, particularly large-scale cohort studies, is crucial to establish a definite association, adjust for potential confounders, and verify the existence of a dose–response relationship.

### Supplementary Information

**Additional file 1.**

**Additional file 2.**

## Data Availability

Data sharing is not applicable to this article as no datasets were generated or analyzed in this study.

## Abbreviations

CO-Hb: Carbon monoxide-hemoglobin
CNS: Central nervous system
GRADE: Grading of Recommendations, Assessment, Development, and Evaluations
nACHRs: Nicotinic acetylcholine receptors
OR: Odds ratio
PRISMA: Preferred Reporting Items for Systematic Reviews and Meta-Analyses
