# Supplementary material for: Exploring the Smoking-Epilepsy Nexus: a systematic review and meta-analysis of observational studies: Smoking and epilepsy
Source: BMC Med. 2024 Mar 4;22:91. doi: 10.1186/s12916-024-03307-0 (PMC10910761; doi:10.1186/s12916-024-03307-0)
Supplement: Supplementary file 1 — Additional file 1. [file 12916_2024_3307_MOESM1_ESM.docx]

**Supplementary table 1.** Search strategy for record extraction

| **Database** | **Search strategy** |
| --- | --- |
| MEDLINE-Embase* | smok*:ab,ti OR tobacco:ab,ti OR nicoti*:ab,ti OR ciga*:ab,ti OR pipe:ab,ti  AND  seizure*:ab,ti OR epileps*:ab,ti OR convulsion*:ab,ti)  AND  cohort:ab,ti OR 'case control':ab,ti OR longitudinal:ab,ti OR 'follow up':ab,ti OR prospective:ab,ti OR retrospective:ab,ti OR incidence:ab,ti  AND  [article]/lim OR [article in press]/lim) AND [humans]/lim |
| Web of Science | TI=(smok*) OR AB=(smok*) OR TI=(tobacco) OR AB=(tobacco) OR TI=(nicoti*) OR AB=(nicoti*) OR TI=(ciga*) OR AB=(ciga*) OR TI=(pipe) OR AB=(pipe)  AND  TI=(seizure*) OR AB=(seizure*) OR TI=(epileps*) OR AB=(epileps*) OR TI=(convulsion*) OR AB=(convulsion*)  AND  TI=(case control) OR AB=(case control) OR TI=(cohort) OR AB=(cohort) OR TI=(longitudinal) OR AB=(longitudinal) OR TI=(follow-up) OR AB=(follow-up) OR TI=(prospective) OR AB=(prospective) OR TI=(retrospective) OR AB=(retrospective) OR TI=(incidence) OR AB=(incidence) |
| Scopus | TITLE-ABS-KEY(smoke) OR TITLE-ABS-KEY(smoking) OR TITLE-ABS-KEY(smoker) OR TITLE-ABS-KEY(smokers) OR TITLE-ABS-KEY(tobacco) OR TITLE-ABS-KEY(nicotine) OR TITLE-ABS-KEY(cigarette) OR TITLE-ABS-KEY(pipe)  AND  TITLE-ABS-KEY(seizure) OR TITLE-ABS-KEY(epilepsy) OR TITLE-ABS-KEY(convlusion)  AND  TITLE-ABS-KEY(case control) OR TITLE-ABS-KEY(cohort) OR TITLE-ABS-KEY(longitudinal) OR TITLE-ABS-KEY(prospective) OR TITLE-ABS-KEY(follow-up) OR TITLE-ABS-KEY(retrospective) OR TITLE-ABS-KEY(incidence) |
| ScienceDirect | smoker OR smoking  AND  seizure OR epilepsy OR  AND cohort OR case-control OR longitudinal OR follow-up |

* The Embase database was utilized for the search as it encompasses the search conducted in MEDLINE.

**Supplementary table 2.** Risk of bias assessment for included studies – Newcastle-Ottawa Scale

| Cohort | Selection | | | | Comparability based on design and analysis | Outcome | | | | Assessment* |
| --- | --- | --- | --- | --- | --- | --- | --- | --- | --- | --- |
|  | Representativeness of the sample | Selection of the non-intervention cohort | Ascertainment of exposure | Demonstration that outcome of interest was not present at start of study |  | Assessment of outcome | Was follow up long enough for outcomes to occur | | Adequacy of follow up of cohorts |  |
| Gao S  (2008) | 1 | 1 | 1 | 1 | 2 | 1 | 1 | | 0 | Good |
| Hamidou B  (2013) | 1 | 1 | 1 | 0 | 2 | 1 | 1 | | 0 | Fair |
| Reiter S.F (2013) | 1 | 1 | 1 | 1 | 2 | 1 | 1 | | 1 | Good |
| Johnson, EL  (2018) | 1 | 1 | 0 | 1 | 2 | 1 | 1 | | 1 | Good |
| Case-control | Selection | | | | Comparability based on design and analysis | Outcome | | | | Assessment* |
|  | Is the case definition adequate? | Representativeness of cases | Selection of Controls | Definition of Controls |  | Assessment of exposure | Same method of ascertainment for cases and controls | | Non-response rate |  |
| Cockerell (1996) | 1 | 0 | 1 | 1 | 2 | 1 | 1 | | 0 | Good |
| Janszky  (2009) | 1 | 0 | 1 | 1 | 2 | 1 | 1 | | 0 | Fair |
| Borthen (2011) | 1 | 0 | 0 | 1 | 1 | 0 | 1 | | 1 | Fair |
| Naldi (2013) | 1 | 1 | 1 | 0 | 2 | 0 | 1 | | 1 | Good |
| Im (2016) | 1 | 0 | 0 | 1 | 1 | 1 | 1 | | 1 | Fair |
| Aguirre  (2017) | 0 | 1 | 0 | 1 | 2 | 0 | 1 | | 1 | Fair |
| Wang  (2021) | 1 | 0 | 1 | 1 | 1 | 0 | 1 | | 1 | Good |
| Cross-sectional | Selection | | | | Comparability based on design and analysis | Outcome | | | | Assessment** |
|  | Representativeness of the sample | Sample size | Non-respondents | Ascertainment of the exposure |  | Assessment of outcome | | Statistical test | |  |
| Kobau (2008) | 1 | 1 | 0 | 1 | 2 | 1 | | 0 | | Satisfactory |
| Svalheim (2013) | 0 | 1 | 1 | 2 | 1 | 1 | | 1 | | Good |
| Cui (2015) | 1 | 1 | 0 | 2 | 0 | 1 | | 1 | | Satisfactory |
| Tumay (2015) | 0 | 1 | 0 | 1 | 1 | 1 | | 0 | | Satisfactory |
| Wang (2016) | 1 | 1 | 1 | 0 | 1 | 0 | | 1 | | Satisfactory |
| Stefanidou  (2022) | 1 | 1 | 0 | 1 | 1 | 2 | | 1 | | Good |

* Good quality: 3 or 4 points in selection domain AND 1 or 2 points in compatibility domain AND 2 or 3 points in outcome/exposure domain, Fair quality: 2 points in selection domain AND 1 or 2 points in comparability domain AND 2 or 3 points in outcome/exposure domain, Poor quality: 0 or 1 point in selection domain OR 0 points in comparability domain OR 0 or 1 point in outcome/exposure domain

** Very good quality: 9-10 points, Good quality: 7-8 points, Satisfactory quality: 5-6 points, Unstatisfactory quality: 0-4 points
