# Supplementary material for: Exploring the Smoking-Epilepsy Nexus: a systematic review and meta-analysis of observational studies: Smoking and epilepsy
Source: BMC Med. 2024 Mar 4;22:91. doi: 10.1186/s12916-024-03307-0 (PMC10910761; doi:10.1186/s12916-024-03307-0)
Supplement: Supplementary file 2 — Additional file 2. [file 12916_2024_3307_MOESM2_ESM.docx]

**Supplementary figure 1.** The forest plot depicting the pooled odds ratio of epilepsy in current smokers compared to non-smokers


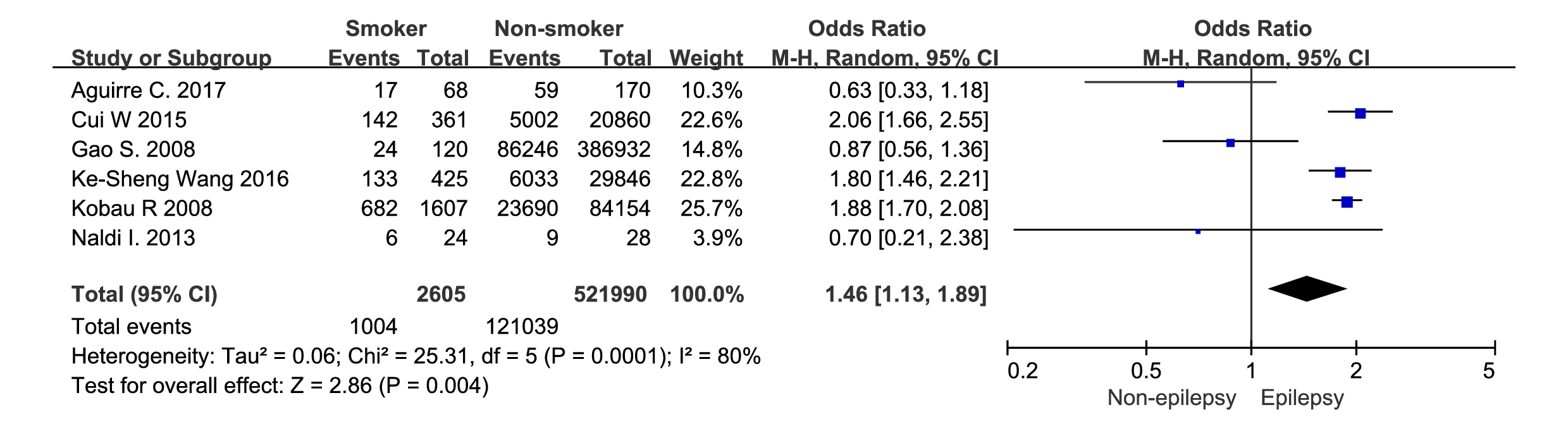


**Supplementary figure 2.** The forest plot depicting the pooled odds ratio of epilepsy in former smokers compared to non-smokers


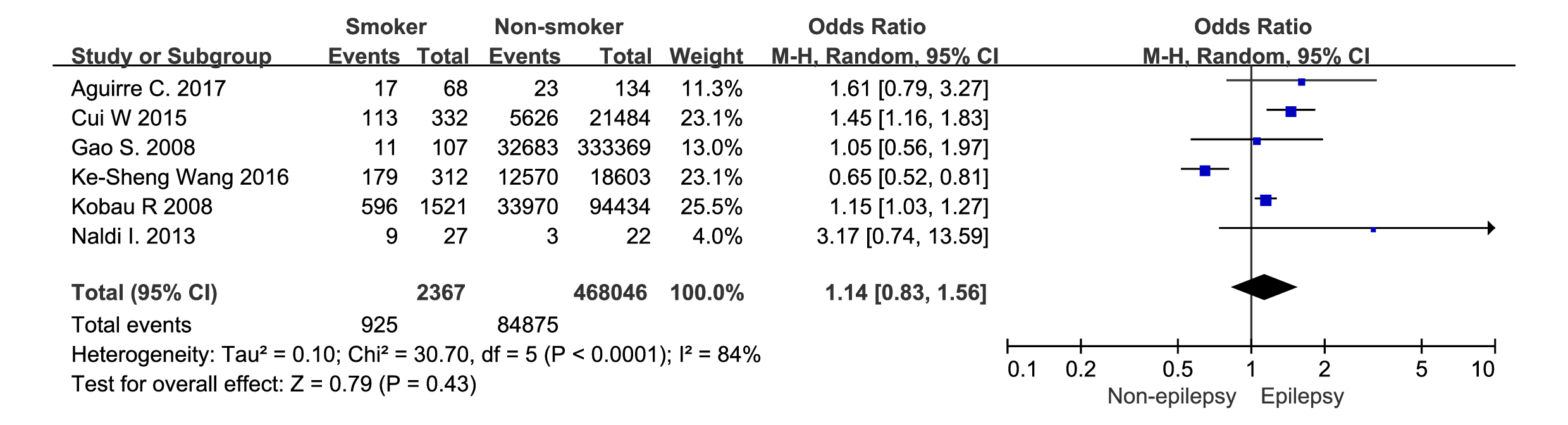


**Supplementary figure 3.** The forest plot depicting the pooled odds ratio of epilepsy in male smokers compared to male non-smokers


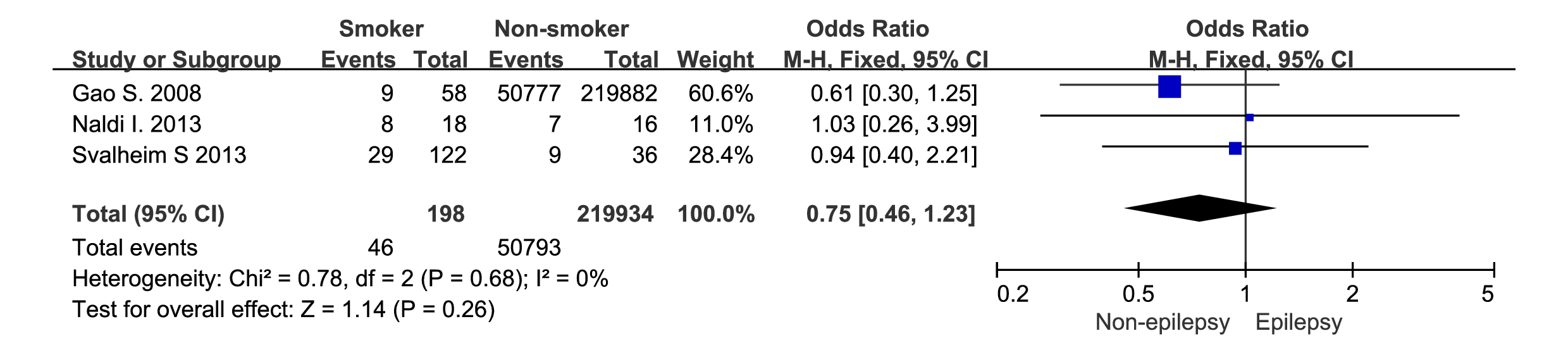


**Supplementary figure 4.** The forest plot depicting the pooled odds ratio of epilepsy in female smokers compared to female non-smokers

**
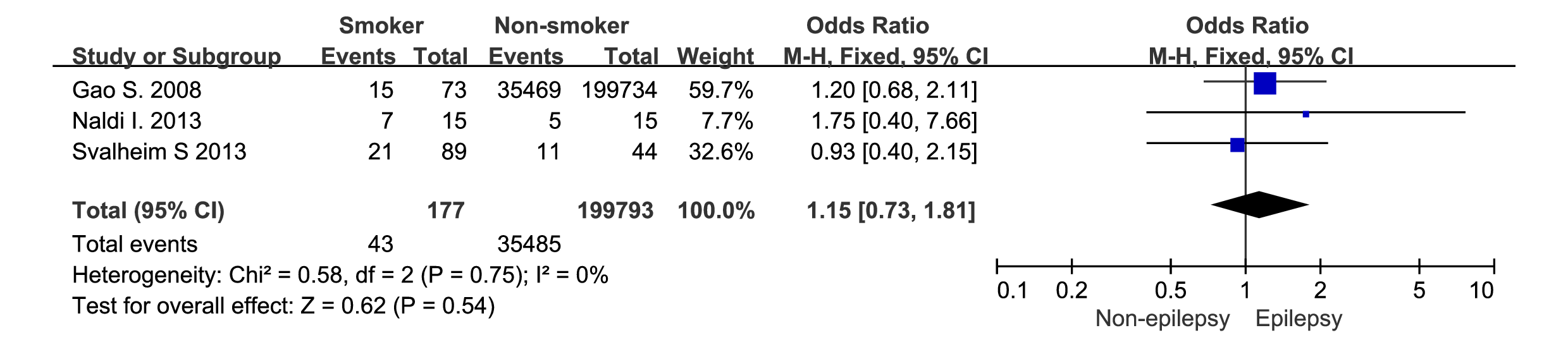
**

**Supplementary figure 5.** The forest plot depicting the pooled odds ratio of epilepsy in smokers compared to non-smokers derived from cohort studies

**
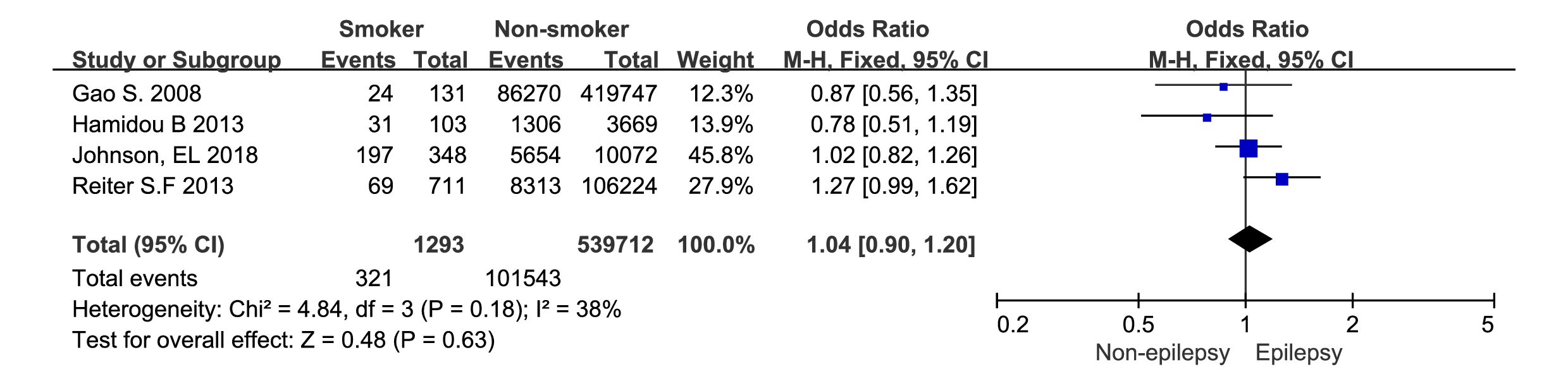
**

**Supplementary figure 6.** The forest plot depicting the pooled odds ratio of epilepsy in smokers compared to non-smokers derived from case-control studies

**
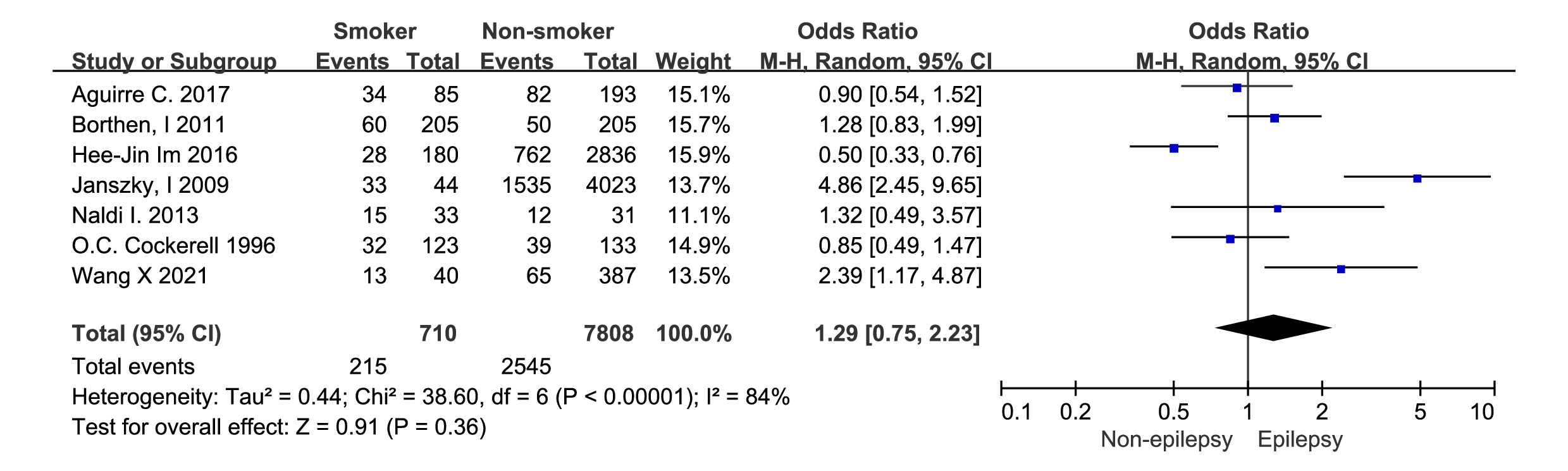
**

**Supplementary figure 7.** The forest plot depicting the pooled odds ratio of epilepsy in smokers compared to non-smokers derived from cross-sectional studies

**
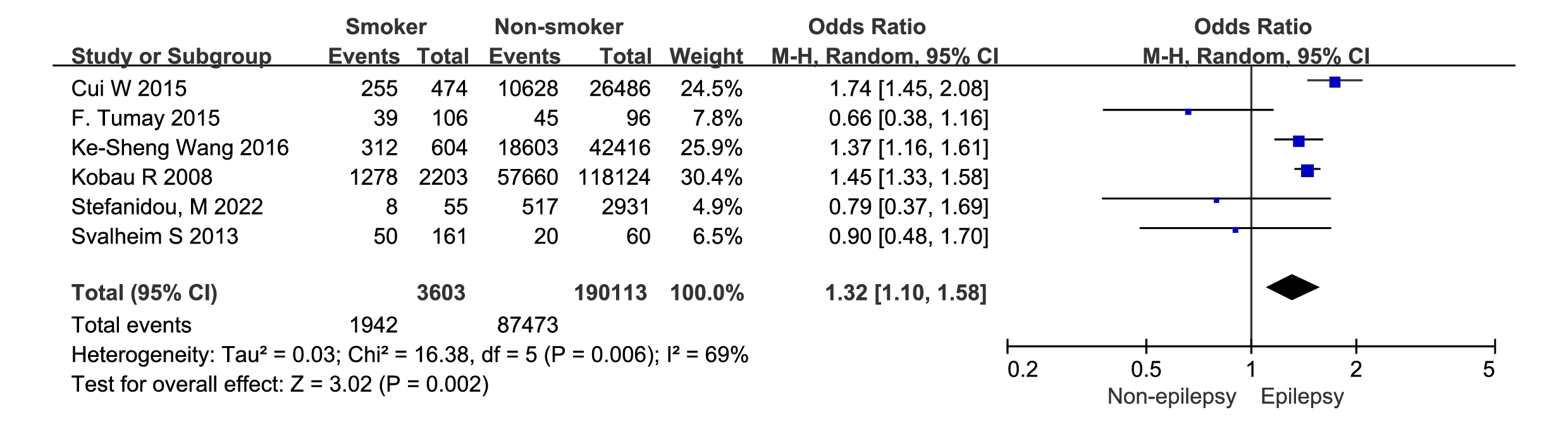
**

**Supplementary figure 8.** The forest plot depicting the pooled odds ratio of active epilepsy in smokers compared to non-smokers

**
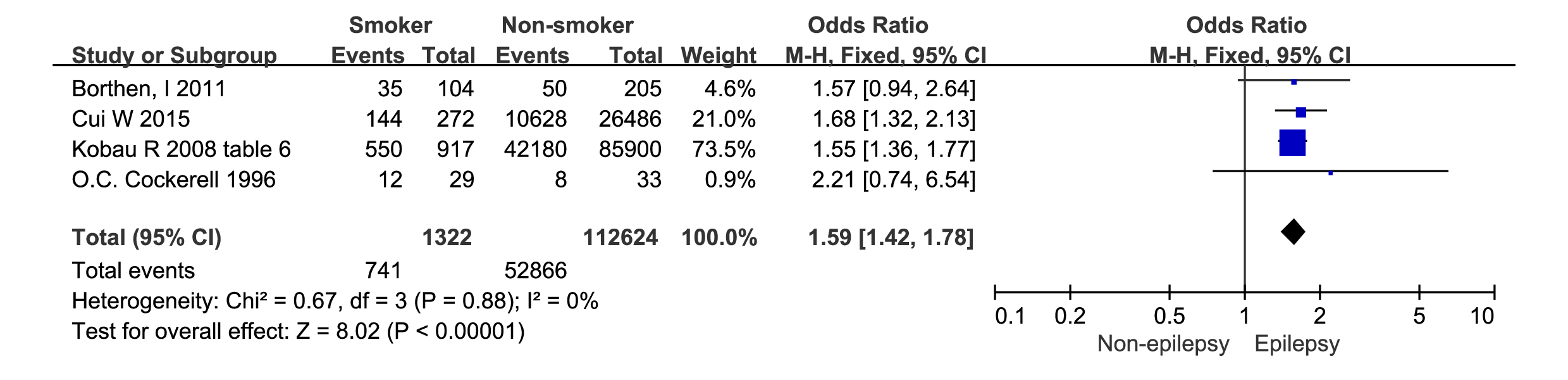
**

**Supplementary figure 9.** The forest plot depicting the pooled odds ratio of active epilepsy in smokers compared to non-smokers

**
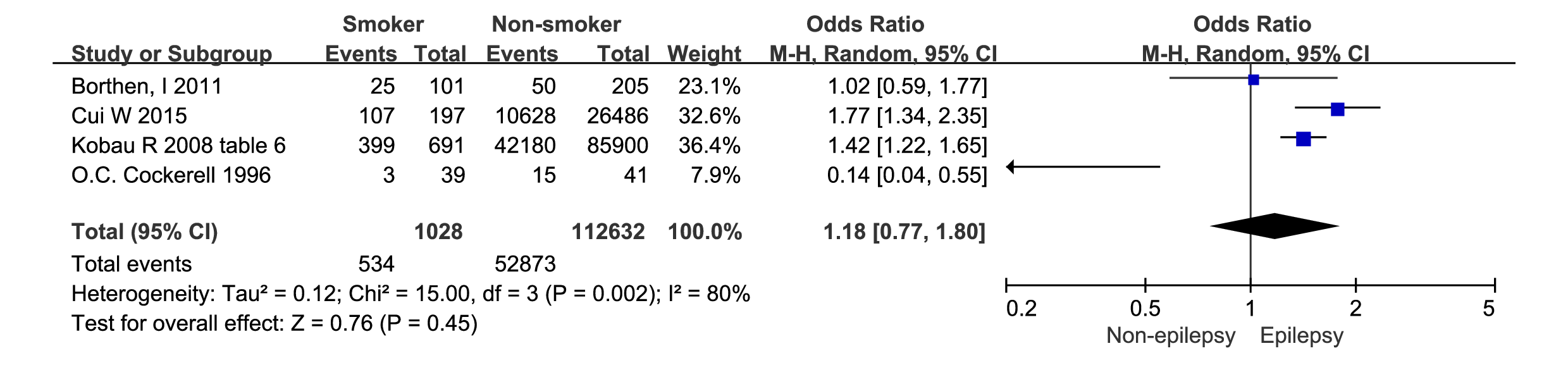
**

**Supplementary figure 10.** The forest plot depicting the pooled odds ratio of epilepsy in smokers compared to non-smokers, with sensitivity analysis excluding the study by Im et al. (2016)

**
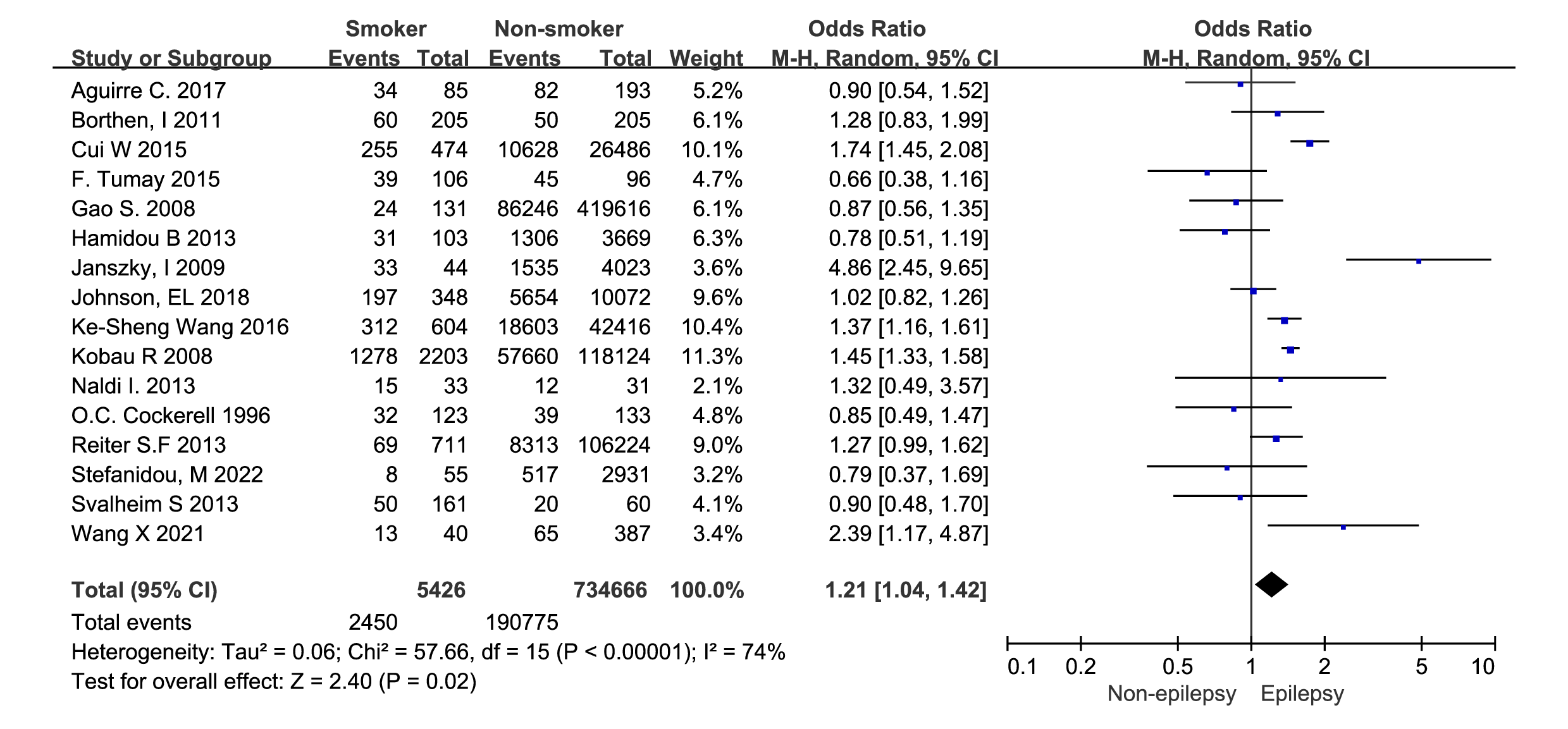
**
